# The use of real-time polymerase chain reaction and an adenosine deaminase assay for diagnosing pleural tuberculosis

**DOI:** 10.4102/ajlm.v8i1.731

**Published:** 2019-08-28

**Authors:** Mulalo Molaudzi, Julitha Molepo

**Affiliations:** 1 Department of Oral Biological Sciences, University of the Witwatersrand, Johannesburg, South Africa

**Keywords:** Pleural tuberculosis, adenosine deaminase assay, qPCR, real-time polymerase chain reaction

## Abstract

**Background:**

The diagnosis of pleural tuberculosis remains a challenge, because the most widely used conventional diagnostic tools are unable to rapidly detect *Mycobacterium tuberculosis* in pleural fluid with sufficient sensitivity.

**Objectives:**

The aim of this study was to evaluate the usefulness of an adenosine deaminase assay and real-time polymerase chain reaction (qPCR) in diagnosing pleural tuberculosis.

**Methods:**

One hundred and five consecutive pleural fluid specimens collected between August 2008 and March 2009 were assessed. Among the 105 specimens, 50 (48%) were unconfirmed tuberculosis cases, 21 (20%) were confirmed tuberculosis cases and 34 (32%) were non-tuberculosis cases (controls). Real-time PCR was performed using the Light Cycler Mycobacterium detection kit according to the manufacturer‘s instructions (Roche Diagnostics). An adenosine deaminase assay was carried out using a commercial colorimetric assay kit as a user-defined method on a Beckman DxC 600 Synchron analyser.

**Results:**

The sensitivity of the qPCR was 67% and specificity was 100%. The sensitivity of the adenosine deaminase assay was 80% and specificity was 94%.

**Conclusion:**

The findings show that the adenosine deaminase assay had higher sensitivity than qPCR. Real-time PCR had 100% specificity, thus a combination of the two methods may be useful for the diagnosis of pleural tuberculosis.

## Introduction

Extrapulmonary tuberculosis is common in South Africa and is exacerbated by the high rate of HIV infection in the country. In 2014 a high incidence of extrapulmonary tuberculosis (88.6 per 100 000 population) was reported in South Africa.^1^ Pleural tuberculosis is the most common form of extrapulmonary tuberculosis after lymph node tuberculosis.^2^ Early diagnosis is essential in order to effectively treat and control the disease. Due to the paucibacillary nature of pleural tuberculosis, the sensitivity of pleural fluid microscopy (about 10%) and culture (about 20%) in pleural tuberculosis cases is low.^3,4,5^ Culture also has a longer turn-around time, ranging from 4 to 8 weeks.^6^

The poor sensitivity and specificity of the conventional methods of diagnosis suggest the need for more effective methods, such as an adenosine deaminase assay (ADA) and real-time polymerase chain reaction (qPCR). Pleural fluid ADA has been shown to be a valuable biochemical marker that has a high sensitivity and specificity for pleural tuberculosis diagnosis.^7^ The use of ADA as a diagnostic marker has additional merits, since results can be produced rapidly.^8^

Molecular diagnostic tests such as qPCR have been used in the diagnosis of tuberculosis. However, the sensitivity of qPCR varies, because different methods target different genes such as *rpoB*, 16s rRNA and *IS6110*.^6,9^ Advantages of qPCR over conventional PCR (polymerase chain reaction)testing include fast availability of results, decreased risk of contamination and quantification of bacterial load.^10^ It is important to find a rapid and reliable test for the diagnosis of pleural tuberculosis, particularly in developing countries such as South Africa where there is high incidence of tuberculosis and HIV. Although the utility of ADA and qPCR for the diagnosis of pleural tuberculosis has been reported worldwide, there is a need to evaluate these tests in developing countries with a high tuberculosis burden.^11^ This study evaluated the use of ADA and qPCR for the diagnosis of pleural tuberculosis.

## Methods

### Ethical considerations

The study was approved by the University of Limpopo (Medunsa campus) Research and Ethics Committee (study approval number: MREC/P/177/2008:PG).

### Study population

One hundred and five consecutive pleural fluid specimens submitted to the Dr George Mukhari Tertiary Chemical Pathology Laboratory in Pretoria, South Africa, between August 2008 and March 2009 were included in the study. The specimens were from 71 suspected tuberculosis cases (patients with suggestive symptoms of tuberculosis; symptoms included coughing for more than 2 weeks, night sweats, fever and loss of weight) and 34 non-tuberculosis cases/controls (patients with other clinical causes of pleural effusion). Pleural fluid of all suspected tuberculosis cases (*n* = 71) and 32 of the control patients were exudates and only 2 of the control specimens were transudates. Among the two patients with transudates, one was diagnosed with malignancy and the other one was diagnosed with diabetes. Patient demographic data, including age, sex and clinical history, were collected from the National Health Laboratory Services Data Intensive System and Applications database. Permission to use samples and access the database was granted by the Head of Chemical Pathology. Individual informed consent was not sought, because the study was conducted on routine samples only and it did not involve additional samples or change in the treatment of patients.

### Clinical and laboratory diagnosis

All pleural fluid specimens were stained using auramine O and cultured on Lowenstein-Jensen agar slants and MGIT BACTEC tubes containing Middlebrook medium (Becton Dickinson, Sparks, Maryland, United States). All the culture positive samples were confirmed by Ziehl-Neelsen staining. A diagnosis of pleural tuberculosis was made when the pleural fluid was culture positive and acid fast bacilli (AFB)-positive for *Mycobacterium tuberculosis*. The unconfirmed pleural tuberculosis cases were diagnosed by the clinical presentation, such as history of tuberculosis infection, chronic cough, night sweats and loss of weight. The non-tuberculosis pleural effusion cases were patients with pleural effusion caused by other medical conditions (Table 1).

**TABLE 1:** Demographic characteristics of cases included in the study, Pretoria, South Africa, 2008-2009.

Study group	Men		Women		Age range (years)	Mean age
	*n*	%	*n*	%		
Confirmed TB cases (*n* = 21)	11	52	10	48	20–65	38.5
Unconfirmed TB cases (*n* = 50)	25	50	25	50	19–85	38.9
Non-TB cases (*n* = 34)	15	44	19	56	23–78	49.0
Malignancy (*n* = 13)	5	38	8	62	30–69	49.2
Congestive cardiac failure (*n* = 9)	3	33	6	67	43–78	63.7
Chronic renal failure (*n* = 5)	3	60	2	40	23–54	34.6
Pneumonia (*n* = 2)	0	0	2	100	27–55	41.0
Polyarthritis (*n* = 2)	2	100	0	0	25–45	35.0
Diabetic (*n* = 2)	1	50	1	50	38–52	45.0
Anaemia (*n* = 1)	1	100	0	0	38	38.0

Confirmed tuberculosis cases: Clinical tuberculosis symptoms and *Mycobacterium tuberculosis* culture positive.

Unconfirmed: Clinical tuberculosis symptoms and *Mycobacterium tuberculosis* culture-negative.

Non-tuberculosis/control cases: Cases with pleural effusion caused by other medical conditions.

TB, tuberculosis.

### DNA extraction

Pleural fluid samples were concentrated by centrifugation at 3000 g for 15 min, the supernatant was discarded and the pellet resuspended in 2 mL of phosphate buffered saline buffer pH 6.8. The samples were mixed by vortexing for 5 seconds. The extraction of DNA was performed on concentrated pleural fluid specimens using the Amplicor respiratory sample preparation kit according to the manufacturer’s instructions (Roche Diagnostics, Mannheim, Germany). Briefly, 500 *µ*L wash solution was added to 100 *µ*L of concentrated pleural fluid sample in a 1.5 mL tube. The mixture was vortexed for 5 s, centrifuged at 12 500 g for 10 minutes, and the supernatant discarded. About 100 *µ*L of lysis buffer was added to the pellet and the tubes were vortexed for 5 s to resuspend the pellet. The samples were then incubated at 60 °C for 45 min. Following incubation, the samples were spun down for 5 s, 100 *µ*L of neutralising buffer was added and the mixture was vortexed for 5 s. The lysate was stored at 2 °C – 8 °C for later use.

### Real-time polymerase chain reaction

Real-time PCR was performed using the Light Cycler Mycobacterium detection kit according to the manufacturer’s instructions (Roche Diagnostics, Mannheim, Germany). A total of 20 *µ*L PCR mixture containing 0.25 *µ*L of 8.5 uracil DNA glycosylase, 0.75 *µ*L internal control, 4 *µ*L master mix, 11 *µ*L detection mix and 4 *µ*L DNA sample was prepared in a capillary tube. An internal control was used to control for the presence of inhibitors. The master mix contained the enzyme Hot Start Taq polymerase, MgCl_2_, deoxynucleoside triphosphate (dTTP, dCTP, dATP, dGTP) and PCR buffer. The detection mix contained the primers, hybridising probes, fluorescein and the acceptor probe LightCycler Red. The primers targeted the 16s rRNA sequence of the *M. tuberculosis* complex. Real-time PCR was performed on the Light Cycler 2.0 (Roche Diagnostics, Mannheim, Germany) real-time instrument according to the following protocol: incubation at 40 °C for 10 min, denaturation at 95 °C for 10 min; amplification consisted of 45 cycles of: 95 °C for 10 s, 50 °C for 10 s with a single acquisition mode and 72 °C for 20 s. This was followed by a melting curve analysis to determine melting temperatures for the sequences targeted by the hybridisation probes. This comprised three stages: 95 °C for 1 min, 40 °C for 2 min and 70 °C for 1 min, ramp rate 0.1 and a continuous acquisition mode, followed by cooling at 40 °C for 30 s.

Analysis of the samples was done in two steps: PCR amplification, where the target amplicon for each sample was detected between the annealing and elongation steps as sigmoid curves at 640 nm. The melting temperature calling, where the melting temperature specific for each subtype in the sample, was determined using the Light Cycler 2.0 software (Roche Diagnostics, Mannheim, Germany). Samples were regarded positive for mycobacteria if they had exponential amplification with a crossing point value of less than 35 with a signal intensity of more than 0.02 and negative with no amplification on the 640/back 530 nm channel and exponential amplification for the internal control in the 705/back 530 nm channel having a signal intensity of more than 0.02. A melting temperature of 53.5 °C – 56.5 °C indicated *M. tuberculosis*, 48 °C – 51 °C *M. avium* and 57 °C – 60 °C *M. kansasii*.

### Adenosine deaminase assay

The ADA was analysed using a commercial colorimetric assay kit according to manufacturer’s instructions (Diazyme General Atomics, Poway, California, United States). The activity in the patient’s specimen was calculated with the formula: activity in specimen = (optical density specimen – optical density specimen blank)/(optical density standard – optical density reagent blank) × 50 with the result expressed in units/L. An ADA value of 30 units/L or higher was considered to be positive. The assay was done on a DxC 600 Synchron analyser (Beckman Coulter, Brea, California, United States) using user-defined methods.

### Data analysis

The results of the ADA assay and qPCR against the culture, the gold standard for diagnosis of tuberculosis, were entered into 2 × 2 tables, wherein the sensitivity was calculated by using the formula TP/(TP+FN), specificity by using the formula TN/(FP+TN), positive predictive value by using the formula TP/(TP+FP) and negative predictive value by using the formula TN/(TN+FN), where *TP* = true positives, *FN* = false negatives, *FP* = false positives and *TN* = true negatives. The mean and standard deviation of ADA and the mean age were calculated using Epi Info version 3.3 (Centers for Disease Control and Prevention, Atlanta, Georgia, United States).

## Results

Among the 105 specimens, 50 (48%) were unconfirmed tuberculosis cases, 21 (20%) were confirmed tuberculosis cases and 34 (32%) were non-tuberculosis cases (controls) (Table 1). All of the 34 (100%) controls were AFB negative, and none showed visible growth on culture medium. Of the 71 suspected tuberculosis cases, 50 (70%) were culture-negative and 21 (30%) were culture positive (i.e. confirmed tuberculosis cases). Of the 21 culture positive samples, 3 (14%) were AFB positive.

Of the 21 confirmed tuberculosis (pleural tuberculosis) cases, 52% were men and 48% were women, with age ranging from 20 to 65 years and a mean age of 38.5 years (Table 1). Of the 50 unconfirmed tuberculosis cases, 50% were men and 50% were women, with age ranging from 19 to 85 years and a mean age of 39 years. Of the 34 non-tuberculosis pleural effusion cases, 38% (13/34) had malignancy, 26% (9/34) had congestive cardiac failure, 15% (5/34) had chronic renal failure, 6% (2/34) were diabetic, 6% (2/34) had pneumonia, 6% (2/34) had polyarthritis and 3% (1/34) had anaemia. Among the 34 control patients, 56% (19/34) were women and 44% (15/34) were men, and their ages ranged from 23 to 78 years, with a mean age of 49 years.

### Real-time polymerase chain reaction

Of the 21 confirmed cases, 14 (67%) were qPCR and culture positive, while 7 (33%) were qPCR negative. None of the specimens from the control group and the unconfirmed tuberculosis cases were positive by qPCR. The sensitivity of the qPCR was 67% and specificity was 100% (Table 2). The positive predictive value was 78% and negative predictive value was 86%.

**TABLE 2:** Operating characteristics of real-time polymerase chain reaction and adenosine deaminase assay in comparison with the gold standard culture in pleural tuberculosis, Pretoria, South Africa, 2008-2009.

Study test	Sensitivity (%)	Specificity (%)	PPV (%)	NPV (%)
qPCR	67	100	78	86
ADA	80	94	89	88

ADA, adenosine deaminase assay; NPV, negative predictive value; PPV, positive predictive value; qPCR, real-time polymerase chain reaction.

### Adenosine deaminase assay

Of the 21 confirmed cases, 17 (81%) were ADA and culture positive, and 4 (19%) were ADA negative. Of the 50 unconfirmed cases, 39 (78%) were ADA positive and 11 (22%) were ADA negative. The ADA levels among the confirmed pleural tuberculosis cases ranged from 8 units/L to 134.3 units/L with a mean ADA value of 52.2 ± 21.66 units/L. The ADA levels among the unconfirmed pleural tuberculosis cases ranged from 5.68 units/L to 200 units/L with a mean ADA value of 50.12.2 ± 21.77 units/L. The ADA levels among the control group ranged between 2.4 units/L and 98 units/L, with a mean ADA value of 12.7 ± 8.64 units/L. Only two (6%) of the 34 non-tuberculosis pleural effusion cases were ADA positive with values of 39 units/L and 98 units/L. Both patients with elevated levels of ADA had pneumonia. The mean ADA level among the pleural tuberculosis cases was significantly higher than that of the control group, *p* < 0.0001. The sensitivity of ADA was 80% and specificity was 94% (Table 2). The positive predictive value was 89% and negative predictive value was 88%.

## Discussion

The diagnosis of pleural tuberculosis is challenging due to the low number of bacilli in the pleural fluid. The detection of pleural tuberculosis using AFB microscopy and culture methods is not effective as both of these methods have low sensitivity, and the culture method has a longer turn-around time of up to 8 weeks.^2,12^

This study evaluated the use of qPCR and ADA for the diagnosis of pleural tuberculosis. The sensitivity and specificity of qPCR varies depending on the type of test used.^13,14,15^ In this study, the sensitivity of qPCR against culture was found to be 67%. Our findings are similar to those of previous studies.^13,14,16^ Rosso et al.^17^ reported even lower sensitivity of 42.8%. A high number of low sensitivities for PCR were reported in a meta-analysis study.^13^ The low sensitivity of qPCR may be explained by the low number of bacilli or the presence of inhibitors in the pleural fluid.^18^ A study by Casallas-Rivera et al.^19^ reported qPCR hybridisation probe sensitivity of 66.7%. This low sensitivity makes it difficult to use PCR as a method for ruling out tuberculosis. In contrast to our findings, higher sensitivity of qPCR has been reported. A study by Kalantri et al.^16^ reported 80% sensitivity for qPCR.

A high qPCR specificity of 100% was found in our study, and other authors have reported similar results. A meta-analysis conducted by Pai et al.^13^ reported commercial and in-house PCR methods to have high specificities (98% and 93%), which suggests a potential role of PCR in confirming the diagnosis of pleural tuberculosis. Other studies reported similar results. Recently, Casallas-Rivera et al.^19^ used qPCR to detect pleural tuberculosis in 40 patients and specificity was found to be 93.5%. In another study, a PCR specificity of 93.8% in 87 patients was reported.^20^ It is noteworthy that some of these comparisons were made between qPCR, the method used in this study which detects amplification during the early phases of the reaction, and PCR, which is a conventional/traditional method detecting amplification at the final phase or end-point of the reaction.

Pleural fluid ADA activity has been shown to be a valuable biochemical marker that has a high sensitivity and specificity for pleural tuberculosis diagnosis.^7^ The mean ADA values among the confirmed pleural tuberculosis and unconfirmed cases in our study were 52.2 ± 21.66 units/L and 50.12 ± 21.77 units/L respectively, which were both significantly higher than that of the control group (12.70 ± 8.64 units/L), with a *p* value of less than 0.0001. These results are in accordance with several other studies.^21,22,23^

The current study showed high ADA sensitivity (80%) and specificity (94%) for the diagnosis of pleural tuberculosis. Several previous studies reported contrasting results to ours. In a study by Mo-Lung et al.,^7^ 210 patients with pleural effusion were studied, and higher ADA sensitivity (87.3%) and lower specificity (91.8%) were observed. A study by Zaric et al.^24^ evaluated the diagnostic value of the ADA assay in 121 patients and found a higher sensitivity (89.2%) and a lower specificity (70.4%). Another study evaluated the ADA assay in more than 2000 patients and reported higher sensitivity (93%) and lower specificity (90%).^25^ Kashyapi et al.^26^ showed higher ADA sensitivity (82%) and lower specificity (83%).

ADA levels in non-tuberculous lymphocytic effusions rarely exceed the diagnostic cut-off for tuberculosis, and various ADA cut-off values have been used, ranging from 30 units/L to 50 units/L. In our study, ADA levels were above the cut-off of 30 units/L value in two (5.8%) patients for whom the diagnosis of pleural tuberculosis was ruled out; both the patients had pneumonia with ADA levels of 98 units/L and 39.8 units/L. This is in agreement with one study where high levels of ADA were reported in some cases of parapneumonic effusions and adenocarcinoma.^27^ Lee et al.^28^ measured ADA levels in non-tuberculous lymphocytic effusion and found that ADA values were above the cut-off of 40 units/L in one complicated parapneumonic effusion and two cases of lymphoma. A study by Porcel and Vives^29^ also showed that it is rare that ADA in non-tuberculous lymphocytic effusions is above the cut-off value. In that study, only two of eight patients with high ADA levels had uncomplicated parapneumonic effusion with ADA levels of 58.9 units/L and 40.5 units/L. Jiménez et al.^30^ studied 410 patients with non-tuberculous lymphocytic effusions and ADA levels reached the diagnostic cut-off for tuberculosis (40 units/L) in seven of the 410 cases. Two patients had bronchogenic carcinomas, two had complicated parapneumonic effusions, one had a diagnosis of lymphoma, one had a mesothelioma and one case was idiopathic. Roughly one-third of parapneumonic effusions had ADA levels above 40 units/L.^31^ Differences in ADA activity between tuberculosis and malignancy may be due to differences in T-helper phenotypes or the presence of memory CD4+ cells in tuberculosis.^23^

### Conclusion

The ADA assay has a high sensitivity (80%) and specificity (94%) and hence it is still a useful tool for the diagnosis of pleural tuberculosis. The sensitivity of qPCR was 67% and specificity was 100%. The low sensitivity of qPCR suggests that this test should not be used for excluding a diagnosis of pleural tuberculosis. However, the high specificity of qPCR suggests that a combination of the two methods may be useful for the diagnosis of pleural tuberculosis. Adenosine deaminase assay (ADA) activity in pleural fluid can differentiate between pleural disease due to tuberculosis and effusion due to non-tuberculous lymphocytic effusion. In addition, the ADA result is available on the same day compared to culture, which takes about two weeks. More studies on the diagnostic value of qPCR are needed.
